# 17β‐Oestradiol promotes differentiation of human embryonic stem cells into dopamine neurons via cross‐talk between insulin‐like growth factors‐1 and oestrogen receptor β

**DOI:** 10.1111/jcmm.13090

**Published:** 2017-02-28

**Authors:** Hong Li, Chenyue Ding, Zhi‐liang Ding, Mingfa Ling, Ting Wang, Wei Wang, Boxian Huang

**Affiliations:** ^1^ Center of Reproduction and Genetics Suzhou Hospital Affiliated to Nanjing Medical University Suzhou China; ^2^ Department of Neurosurgery Suzhou Hospital Affiliated to Nanjing Medical University Suzhou China

**Keywords:** 17β‐Oestradiol, human embryonic stem cell, neuronal cell differentiation, neural precursor cell, dopaminergic neuron

## Abstract

Human embryonic stem cells (hESCs) can self‐renew and differentiate into all cell lineages. E2 is known to exhibit positive effects on embryo development. Although the importance of E2 in many physiological processes has been reported, to date few researchers have investigated the effects of E2 on hESCs differentiation. We studied the effects of E2 on dopamine (DA) neuron induction of hESCs and its related signalling pathways using the three‐stage protocol. In our study, 0.1 μM E2 were applied to hESCs‐derived human embryoid bodies (hEBs) and effects of E2 on neural cells differentiation were investigated. Protein and mRNA level assay indicated that E2 up‐regulated the expression of insulin‐like growth factors (IGF)‐1, ectoderm, neural precursor cells (NPC) and DA neuron markers, respectively. The population of hESC‐derived NPCs and DA neurons was increased to 92% and 93% to that of DMSO group, respectively. Furthermore, yield of DA neuron‐secreted tyrosine hydroxylase (TH) and dopamine was also increased. E2‐caused promotion was relieved in single inhibitor (ICI or JB1) group partly, and E2 effects were repressed more stronger in inhibitors combination (ICI plus JB1) group than in single inhibitor group at hEBs, hNPCs and hDA neurons stages. Owing to oestrogen receptors regulate multiple brain functions, when single or two inhibitors were used to treat neural differentiation stage, we found that oestrogen receptor (ER)β but not ERα is strongly repressed at the hNPCs and hDA neurons stage. These findings, for the first time, demonstrate the molecular cascade and related cell biology events involved in E2‐improved hNPC and hDA neuron differentiation through cross‐talk between IGF‐1 and ERβ *in vitro*.

## Introduction

E2 is an important female sex steroid, which is also known as biologically active oestrogen. Previous studies have demonstrated that it is vital for growth, development, differentiation, maturation and function of central nervous system in post‐pubertal mammal 1, including synaptic plasticity and excitability, neuroprotection and survival, and neurotransmitter and neuropeptide synthesis. In addition, E2 has ability to against the neurotoxic effects of glutamate 2 and oxidative stress 3. Animal research revealed that high E2 level administration has been investigated as modalities for cerebral ischaemia 4. It is also important in regulating the activity of non‐neural tissues, such as reproductive system 5 and cardiovascular system 6. Oestrogen receptor comprise two subtypes, oestrogen receptor (ER)α and ERβ. Both of them typically act as transcription factors and regulate ER‐dependent gene expression via the genomic signalling pathway. Concurrently, they are also taking different ways to regulate the aforementioned functions 7. As the report noted, during the fertile period, oestrogen is kept at a high level throughout pregnancy in female bodies 8, which does mean that the level of E2 in gravidae plays a central role in the process of embryo development.

Insulin‐like growth factors (IGFs), both IGF‐1 and IGF‐2, are peptide hormones that have important roles in mammalian growth, development and maturation, but have a much higher growth‐promoting activity than insulin. IGF‐1 binds to the IGF‐1 receptor and provokes intracellular signalling cascades 9. There are accumulating evidence for an abundant expression of IGF‐IR and ER in hippocampus 10, 11. The interaction of IGF‐1 and E2 may promote neuroprotection under neurodegenerative conditions 12. It has been reported that IGF‐1R and oestrogen receptors (ERα and ERβ) are co‐expressed in many neurons and glial cells in the CNS 11. Pioneering work has shown that there is a cross‐talk between IGF‐1R and ER in cancer 13, and our previous paper reported that E2 up‐regulated the expression of IGF‐1 at human embryonic stem cells (hESCs) differentiation stage 14. Although, the neuroprotective effects of IGF‐1 have been previously described, the correlation between IGF‐1 and ER, also their connection with regulation each other in the process of hESCs differentiation into dopamine (DA) neurons, remains unclear.

Human embryonic stem cells were first obtained from the inner cell mass of blastocysts 15. They can self‐renew indefinitely and maintain their pluripotency in culture system. They also possess a long‐term proliferative capacity, making them amenable to cell culture *in vitro,* but they readily generate multiple differentiated three germ layer cell types in culture 16. Recently, vast quantities of scientists suggested that hESCs as a cellular model mimic embryonic development which could be studied under *in vitro* conditions 17. Subsequently, researchers proposed a concept of embryonic stem cell test (EST)^18^, which is an animal‐free method used to assess the embryotoxic potential of reagents *in vitro*. Previous work showed that EST can be used to predict accurately the embryotoxicity of compounds in a multiple fields, such as bisphenol A, ethanol and triclosan 14, 19, 20. In the meanwhile, mRNAs of oestrogen receptor α and receptor β were found to be present in ESCs 21, suggesting that oestrogen may have a role in maintaining the characteristics of ESCs. Therefore, in this study, human embryonic stem cell as a model was used to investigate our hypotheses that whether E2 could promote hESC differentiation in three stages (EB, neural precursor cells (NPC) and DA), whether this effect is mediated through oestradiol receptor (α or β) or IGF‐1.

## Materials and methods

### Cultivation of hESCs

Undifferentiated H9 hESCs (46, XX) from WiCell Research Institute (Madison, WI, USA) were cultured as described previously 16. Medium was changed daily. The hESC culture medium is composed of DMEM/F12, 20% knockout serum, 1% glutamine, 1% non‐essential amino acids, 0.1 mM 2‐mercaptoethanol, 1% penicillin streptomycin (all purchased from Thermo, Waltham, MA, USA) and 10 ng/ml basic fibroblast growth factor (bFGF: Peprotech, Rocky Hill, NJ, USA). Briefly, mouse embryonic fibroblasts (MEFs) were isolated from embryos of 12.5‐day old ICR mouse embryos and treated by mitomycin C (MC). The hESCs were cultured on the MC‐treated MEFs for 5–7 days at 37°C with 5% CO_2_, 20% O_2_. The experimental protocol for MEFs isolation was approved by the Institutional Animal Care Committee of Nanjing Medical University, and the experimental protocol for hESCs culture and differentiation was carried out in accordance with the approved guideline of ‘Experiment Research on Human Embryonic Stem Cell (2008)’ by the Ethics Committee of Nanjing Medical University.

### Differentiation of hESCs into DA neurons

**Figure 1:**
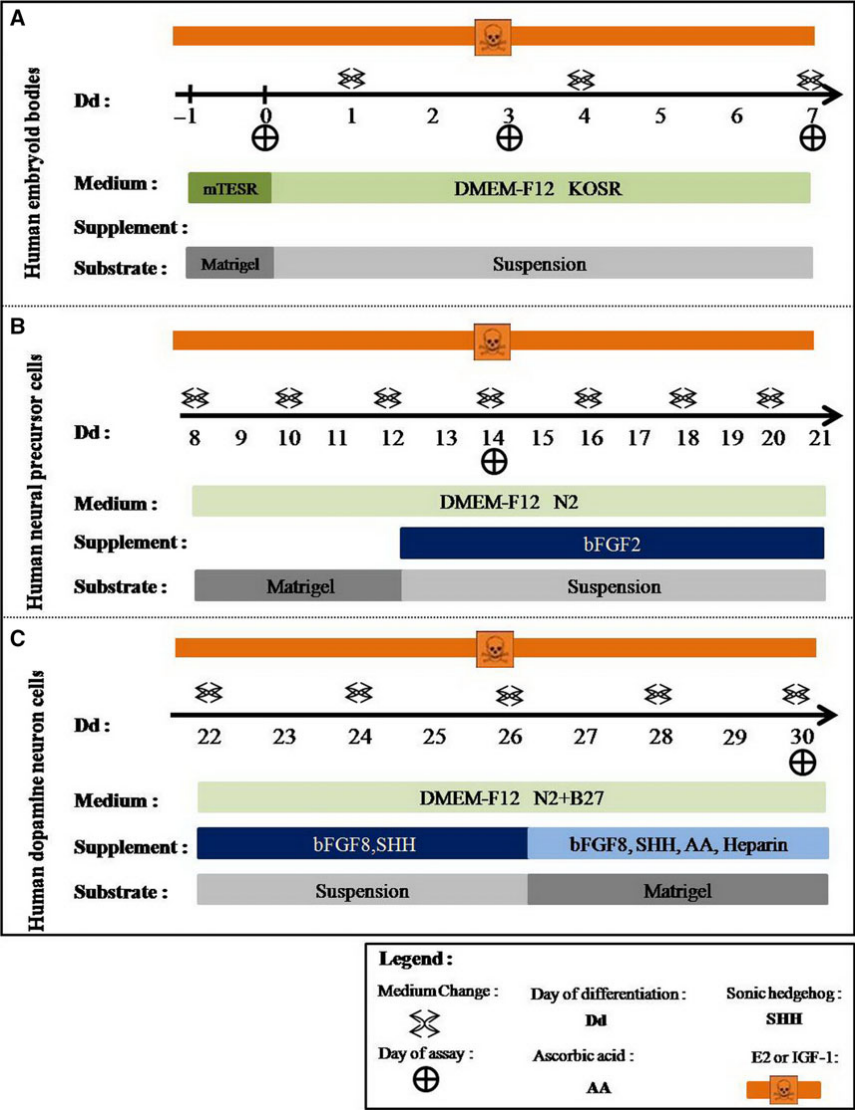
Protocol for differentiation dopamine (DA) neurons from human embryonic stem cells (hESCs). Human embryonic stem cells were induced differentiation into DA neurons through three stages (stage I: embryoid bodies; stage II: induction neural precursor cells (NPCs); stage III: differentiation into DA neurons) according to previous report 14 with slight modification. (**A**) Stage I: hEBs were cultured with normal culture medium without growth factor; (**B**) Stage II: hEBs were differentiated into NPCs by cultured for 5 days on mareigel in the NPCs‐induced medium (composed of 1% N2) and cultured for next 10 days on suspension in the NPCs‐induced medium (composed of 1% N2, 10 ng/ml bFGF‐2). (**C**) Stage III: NPCs were differentiated into DA neurons by cultured for 5 days on suspension in the neuron‐induced medium (composed of B27, 1% N2, 200 ng/ml SHH, 100 ng/ml bFGF‐8) and cultured for next 5 days on matrigel in the neurons‐induced medium (composed of B27, 1% N2, 200 ng/ml SHH, 100 ng/ml bFGF‐8, 200 μM ascorbic acid (AA) and 50 mg/ml heparin). Cells were exposed to 0.1% DMSO, 0.1 μM E2, 0.1 μM E2 plus 1 μM JB1, 0.1 μM E2 plus 2 μM ICI or 0.1 μM, E2 plus inhibitors combination (2 μM ICI plus 1 μM JB1), respectively, during all differentiation stages and steps.

H9 cells were induced differentiation into DA neurons through three stages (stage I: formation human embryoid bodies (hEBs); stage II: induction neural precursors; stage III: differentiation into DA neurons) according to previous report 14 with slight modification (Fig. 1). In stage I, hESC colonies were detached with 1 mg/ml collagenase IV (Thermo) and 1× dispase (Thermo) and then cultured for 7 days to form EBs in ultralow‐attachment dishes containing the basic differentiation media (BDM, hESCs culture medium without bFGF) under suspended conditions. In stage II, EBs were cultured for 4 days to induce NPCs in NPC‐induced medium (composed of BDM and 0.5% N2). And then, to form spherical neural masses (SNMs), the neural rosettes and neural tube‐like structures were mechanically isolated and cultured for another 10 days in ultralow‐attachment dishes containing the NPCs expansion medium (composed of BDM, 1% N2 and 10 ng/ml bFGF). In stage III, SNMs were differentiated into precursor DA neurons by cultured for 4 days in the neuron‐induced medium (composed of B27, 1% N2, 200 ng/ml (Sonic hedgehog, SHH) and 100 ng/ml bFGF‐8). Next, neural cells were differentiated into DA neurons by cultured for another 4 days in the neuron‐induced medium (composed of B27, 1% N2, 200 ng/ml SHH, 100 ng/ml bFGF‐8, 200 μM ascorbic acid (AA) and 50 mg/ml heparin). Cells were exposed to 0.1 μM E2 or 0.1 μM E2 plus IGF‐1 or E2 inhibitor (2 μΜ ICI or 1 μΜ JB1) (ICI = ICI 182, 780, Sigma‐Aldrich, USA; JB1, Sigma‐Aldrich, St. Louis, MO) and 0.1 μM E2 plus inhibitors combination group, respectively, during all differentiation stages, and parallel experiments in IGF‐1 (0.1 μM) treatment group were performed (Fig. 1). Meanwhile, 0.1% dimethylsulphoxide (DMSO, carrier solvent used to dissolve E2) exposure groups were set as controls (Fig. 1).

### RNA extraction and real‐time polymerase chain reaction (PCR)

hESCs total RNA was extracted using the QIAGEN RNeasy Mini Kit (QIAGEN, Valencia, CA, USA) and then was reverse‐transcribed to cDNA by PrimeScript RT Reagent Kit (Takara, Nojihigashi Shiga, Japan) according to the manufacturer's methods, and the total RNA concentration was determined by measuring the absorbance at 260 nm. Quantitative real‐time PCR was carried out with SYBR Premix Ex Taq (Takara) on Thermal Cycler Dice Real Time System (Takara). Real‐time PCR (Applied Biosystems, ABI PRISM 7900, Drive Foster City, CA, USA) was carried out for 40 cycles of denaturation at 95°C for 15 sec., annealing at 58°C for 15 sec. and extension at 72°C for 30 sec. Cycle time (Ct) values were obtained after analysed by the Sequence Detection System and analysis software (Applied Biosystems). 2^−ΔΔCt^ calculation method was used to analyse data. Each sample was quantified against its GAPDH transcript content. Experiments were repeated three times. Results are presented as fold change ± SD, and *P* < 0.05 is determined significant difference. The sequences of used primers are shown in Table 1.

**Table 1:** Designations, sequences and the sizes of real‐time PCR amplicons

Name	Sequence from 5′‐3′	Size (bp)	Acc. number
Nestin Forward	TTGCCTGCTACCCTTGAGAC	145	NM_006617.1
Nestin Reverse	GGGCTCTGATCTCTGCATCTAC		
Sox1 Forward	CCTGTGTGTACCCTGGAGTTTCTGT	180	NM_005986.2
Sox1 Reverse	TGCACGAAGCACCTGCAATAAGATG		
IGF‐1 Forward	ATCAGCAGTCTTCCAACCCAA	209	NM_001111283
IGF‐1 Reverse	AAATAAAAGCCCCTGTCTCCAC		
Musashi‐1 Forward	AGCTTCCCTCTCCCTCATTC	160	NM_002442.3
Musashi‐1 Reverse	GAGACACCGGAGGATGGTAA		
TUJ‐1 Forward	GAGACCTACTGCATCGACAA	151	NM_006086
TUJ‐1 Reverse	CATTGAGCTGACCAGGGAAT		
TH Forward	GTGCTAAACCTGCTCTTCTC	83	NM_199292
TH Reverse	GCTTCAAACGTCTCAAACAC		
GAPDH Forward	GAAGGTCGGAGTCAACGGATTT	223	NM_002046
GAPDH Reverse	CTGGAAGATGGTGATGGGATTTC		

### Western blot analysis

Samples from differentiation days 14 and 30 were harvested and dissociated in a lysis buffer. Protein was extracted from each sample, which was then loaded on 10% gels and separated by SDS‐PAGE (sodium dodecyl sulphate polyacrylamide gel electrophoresis). Next, the separated proteins were transferred to polyvinylidene difluoride membrane (PVDF, Millipore, USA). Thirdly, the proteins were incubated with the primary antibodies (Abcam, Cambridge, MA, USA) of anti‐human‐NESTIN, anti‐human‐TUJ‐1, anti‐human‐tyrosine hydroxylase (TH), anti‐human‐MSI‐1, anti‐human‐Gapdh, anti‐human‐β‐tubulin, anti‐human ERα, anti‐human ERβ and appropriate secondary antibodies (goat anti‐rabbit HRP conjugates; Jackson Immunoresearch, West Grove, PA, USA) separately. The specific signals were detected by the enhanced chemiluminescence (Pierce ECL Western blotting Substrate; Thermo). Finally, the membrane was checked by a chemiluminescence detection system (Tanon, Shanghai, China) and the signal intensity of each band was analysed by Imaging J Software (National Institutes of Health, USA). Experiments were repeated three times, results are presented as fold change ±SD and *P* < 0.05 is determined significant difference (The assay of used antibodies are shown in Table 2).

**Table 2:** The information of antibodies

Antibody name	Company	Catalogue number	Experiment
IGF‐1	Abcam	ab9572	WB
SOX1	Abcam	ab109290	FACS
NESTIN	Abcam	ab6320	WB and FACS
MSI‐1	Abcam	ab52865	WB and FACS
TUJ‐1	Abcam	ab78078	WB and FACS
TH	Abcam	ab75875	WB and FACS
ERα	Abcam	ab32063	WB and FACS
ERβ	Abcam	ab288	WB and FACS

WB, Western blot; FACS, fluorescence‐activated cell sorter; ER, oestrogen receptor; TH, tyrosine hydroxylase; IGF, insulin‐like growth factors.

### Immunofluorescence staining

The primary antibodies of anti‐human‐TUJ‐1 (Abcam) and anti‐human‐TH (Abcam) were selected to characterize differentiated neuronal cells. For the staining procedure, cells cultured on cover slips were fixed with 4% (w/v) paraformaldehyde (PFA; Sigma‐Aldrich) at room temperature for 10 min. and then washed three times for 5 min. with phosphate‐buffered solution (PBS), permeated with 0.1% Triton X‐100 (Sigma‐Aldrich)/PBS on ice for 10 min, and blocked with fresh 4% bovine serum albumin (BSA; Sigma‐Aldrich)/PBS at room temperature for 30 min. The treated cells were washed with PBS three times for 5 min. And then incubated with primary antibodies over night at 4°C. After rinsed with PBS for 5 min, the cells were stained by Cy2‐ or FITC‐conjugated secondary antibodies (Jackson Immunoresearch) in dark at room temperature for 30 min. The stained cells were mounted with 4′, 6‐diamidino‐2‐phenylindole (DAPI; Vector Lab, Burlington, Canada) after washed with PBS for 5 min. And then photographed under fluorescence microscope (Olympus, Tokyo, Japan).

### Fluorescence‐activated cell sorter (FACS) analysis

Differentiated hESCs were digested by trypsin‐EDTA for 3 min. and blown into single cells gently, which were fixed and permeated by the Cytofix/Cytoperm Fixation/Permeabilization Solution Kit (BD, NJ, USA) following the manufacturer's instruction. Treated cells were then stained with PE‐ or FITC‐conjugated antibodies of anti‐human‐NESTIN, anti‐human‐MSI‐1, anti‐human‐TH and anti‐human‐TUJ‐1 or their corresponding isotype control, for 30 min, at 4°C as above described. The stained cells were analysed on fluorescence‐activated cell sorter (Beckman, Boulevard Brea, CA, USA). Experiments were repeated three times, results are presented as fold change ±SD and *P* < 0.05 is determined significant difference.

### Gene silencing with RNA interference

IGF‐1 siRNA (Thermo Fisher, AM16708) and ERβ siRNA (Santa Cruz, sc‐35325) were transfected into cells at the final concentration of 40 nM to silence IGF‐1 and ERβ, respectively, at differentiation day 11 (NPCs stage) when using Dharmafect 1 (Dharmacon, cat. T‐2001‐02) transfection reagent, following the manufacturer's instructions. To plate cells onto a 12‐well before transfection so that they are 50% confluent for transfection, we used 2 μl of transfection reagent, 2 μl of 20 mM siRNA solution and 4 × 10^4^ cells (NPCs stage) in 1 ml of culture medium at differentiation day 11. The efficacy of gene silencing was checked with Western blot analysis and found to be optimal at 72 hrs.

### Enzyme‐linked immunosorbent assay (ELISA) analysis

Suspended culture media from DA neurons differentiation system at days 24, 28 and 30, respectively, was harvested to evaluate the expression level of tyrosine hydroxylase and dopamine decarboxylase using an ELISA kit (Antibodies, Atlanta, GA, USA) according to the manufacture's guide. Briefly, 10 μl old culture media was added into 40 μl sample dilution and mixed gently. The test plate was wrapped with membrane, incubated for 30 min. at 37°C. Thereafter, wells on plate were dried and washed with wash buffer for five times (30 sec. per time). Then 50 μl HRP‐conjugate reagent was added into each sample well and incubated for 15 min. at 37°C. Samples were washed with wash buffer for five times (30 sec. per time). Subsequently, 50 μl number A chromogen solution followed by 50 μl number B chromogen solution were added and incubated for 15 min, at 37°C. Then 50 μl stop solution was added into each control and sample well. Finally, the light absorbance was measured and recorded by a spectrophotometer (Varian Company, North Charleston, SC, USA).

### Statistical analysis

All results were showed as means ± SD. Statistically significant difference was determined by one‐way anova with SPSS 17.0 (Chicago, IL, USA) software, and *P* < 0.05 was regarded as statistical significance.

## Results

### Effects of E2 on colony morphology and cell viability in hEBs

**Figure 2:**
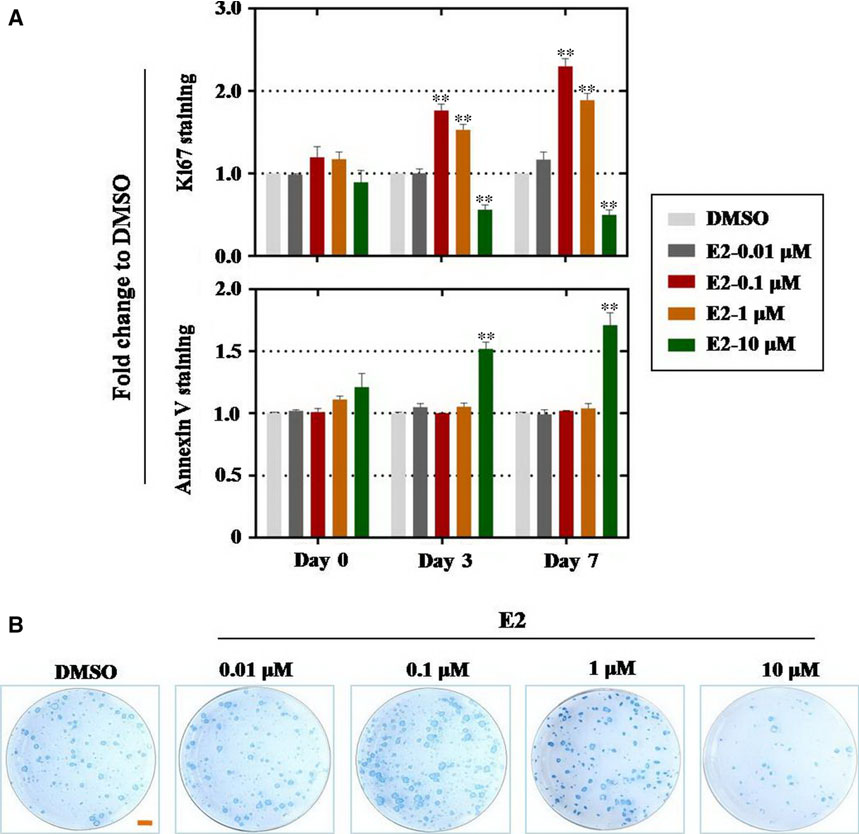
0.1 μM E2 promotes hEBs proliferation in a dose‐ and time‐dependent. (**A**) Proliferation and apoptosis rates of hEBs were evaluated by FACS assay. First panel (Ki67 staining assay): 0.01 μM and E2 had no significant effects on hEBs proliferation, and 0.1 μM and 1 μM E2 significantly increased the proliferation rate of hEBs, 10 μM E2 significantly decreased the proliferation rate of hEBs; second panel (annexin V staining assay): 10 μM BPA significantly increased the early apoptosis rate of hEBs. (**B**) The morphology of hEBs exposed to 0.01 μM E2 was similar to that of DMSO group, while hEBs treated by 0.1 μM and 10 μM E2 increased the number of clones, by high concentration of 10 μM E2 presented abnormal appearance. Scale bar, 100 μm. *n* = 3; error bars indicate SD; *P < 0.05; **P < 0.01; ***P < 0.001.

To examine the effects of E2 on cell proliferation and apoptosis, hEBs were treated to increase the concentration of E2 at day 1, day 3 and day 7 (Fig. 1A). FACS assay was employed to assess quantitatively for cell viability. Our results showed that E2 significantly increased cell viability at 0.1 and 1 μM compared with the control group at day 3 and day 7 (Fig. 2A), in particular 0.1 μM. However, E2 induced suppressive effect when using 10 μM concentration (Fig. 2A). Similarly, the results from apoptosis assay indicated that high‐dose E2 (10 μM) increased the rate of apoptosis at day 3 and day 7 strikingly (Fig. 2A). Alkaline phosphatase staining experiment was executed at differentiation day 7, results manifested that 0.1 μM and 1 μM E2 were advanced the number of hESCs and reverse effect was manifested in 10 μM group (Fig. 2B). Before the effects of E2, hESCs differentiation stage was evaluated. It was confirmed that 0.1 μM E2 was a suitable concentration to exposure.

### E2 treatment up‐regulated ectoderm genes expression through cross‐talk between IGF‐1 and ER

**Figure 3:**
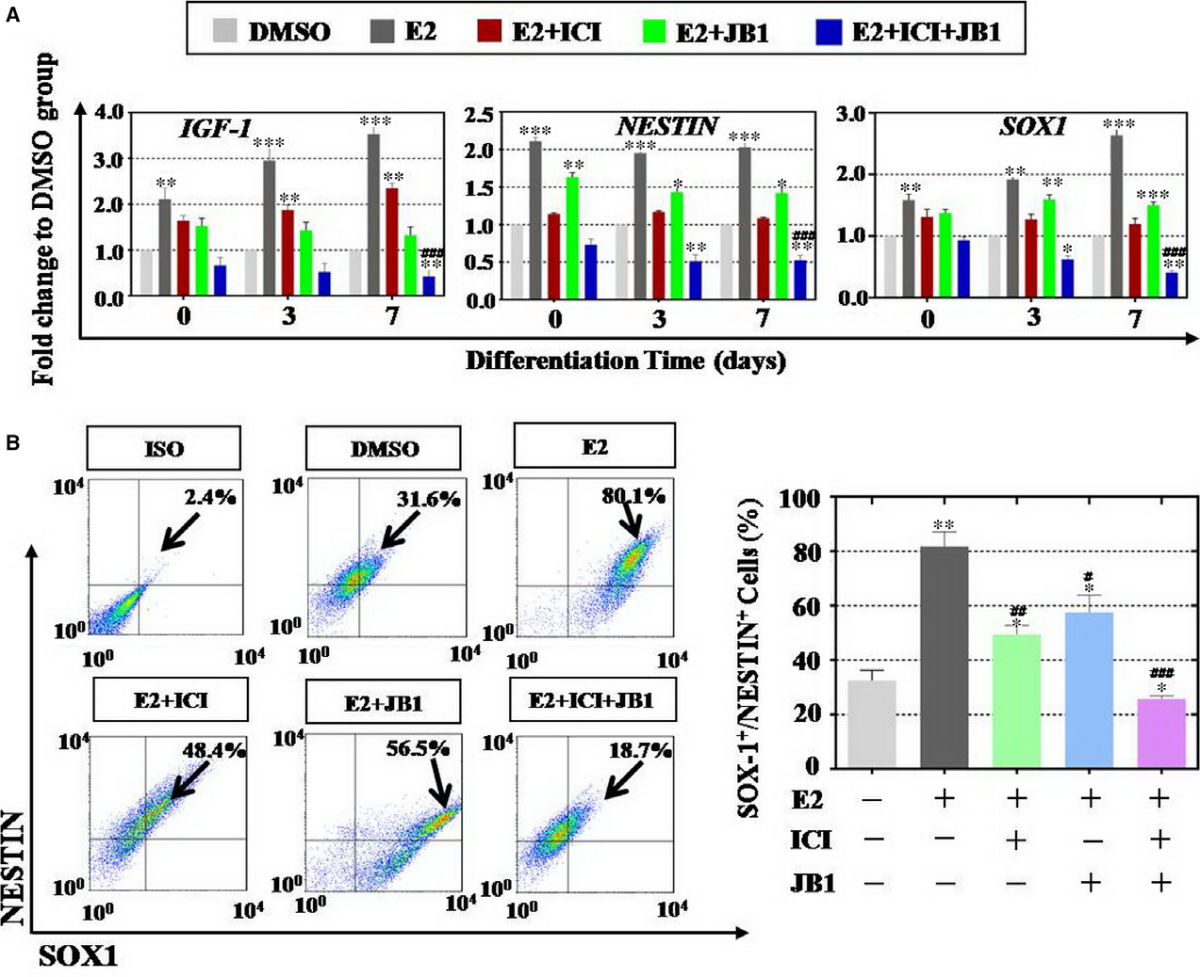
E2 exposure up‐regulated insulin‐like growth factors (IGF)‐1 and marker genes’ expression of ectoderm layers during human embryonic stem cells (hESCs) differentiation period. (**A**) *IGF‐1*,* NESTIN* and *SOX‐1* were measured by qPCR assay at three time‐points. E2 increased the expression of ectoderm markers of *NESTIN* (days 0, 3 and 7), *SOX1* (days 0, 3 and 7) and *IGF‐1* (days 0, 3 and 7), while ICI or JB1 singly applied repressed *NESTIN* (days 0, 3 and 7), *SOX1* (days 3 and 7) and *IGF‐1* (days 0, 3 and 7) expression partially. Inhibitors combination (ICI plus JB1) significantly reduced *NESTIN* (days 3 and 7), *SOX1* (days 3 and 7) and *IGF‐1* (day 7) expression. (**B**) At day 7, IGF‐1, NESTIN and SOX‐1 were measured by FACS assay. Results indicated that one inhibitor just curbed E2 effect slightly, and inhibitors combination strongly decreased the population of NESTIN^+^SOX‐1^+^ cells significantly, *n* = 3; Error bars indicate SD. *P < 0.05, **P < 0.01; ***P < 0.001 (compared with the DMSO group). #*P* < 0.05; ##*P* < 0.01; ###*P* < 0.001 (compared with the E2 group)

To assess effects of E2 on the differentiation of hESCs at hEBs stage, firstly, hEBs were cultured and treated as above described (Fig. 1A). Colonies at differentiation days 0, 3 and 7 were collected, and qPCR method was used to test the expression level of ectoderm markers and IGF‐1. As shown in Figure 3A, E2 could significantly increase mRNA level of *IGF‐1* at all tested three time‐points (211%, 295% and 353%). Similar, ectodermal expression of NESTIN and SOX1 was significantly up‐regulated at day 0 (211% and 158%), day 3 (195% and 192%) and day 7 (203% and 263%) compared to DMSO group (Fig. 3A). However, single inhibitor (ICI or JB1) treatment group showed significantly lower expression of *IGF‐1* (175% or 132%), *NESTIN* (131% or 142%) and *SOX1* (129% or 150%) than E2 group at day 7. Moreover, inhibitors combination group (JB1 plus ICI) implicated that the expression level of *IGF‐1*,* NESTIN* and *SOX1* was greatly decreased to 42%, 52% and 41% at differentiation day 7 compared to DMSO group. IGF‐1 treatment group showed the similar results. As shown in Figure S2A, IGF‐1 administration significantly increased mRNA level of *IGF‐1* (215%, 355%, 453%), *NESTIN* (201%, 295%, 375%) and *SOX1* (152%, 202%, 285%) at all tested three time‐points which compared to DMSO group. Single inhibitor (ICI or JB1) treatment group showed significantly lower expression of *IGF‐1* (255% or 145%), *NESTIN* (181% or 112%) and *SOX1*(189% or 140%) than E2 group at day 7 (Fig. S2A). Meanwhile, inhibitors combination group (JB1 with ICI) implicated that the expression level of *IGF‐1*,* NESTIN* and *SOX1* was greatly decreased to 52%, 60% and 51% at differentiation day 7 compared to DMSO group (Fig. S2A). Undoubtedly, mRNA level of *IGF‐1*,* NESTIN* and *SOX1* in inhibitors combination group was negative expression compared to single inhibitor group. Consistent with qPCR assay results, FACS data confirmed that E2 increased NESTIN^+^SOX1^+^ cell number (82%) at differentiation day 7, and one inhibitor group (ICI or JB1) notably repressed NESTIN^+^SOX1^+^ cells number (48% or 57%), respectively. Two inhibitors combination exposure group at differentiation day 7 indicated that the expression of *NESTIN‐* and *SOX1*‐positive cells was down‐regulated, and only 17% was left to that of DMSO group (Fig. 3B). In FACS assay, IGF‐1 treatment group showed the similar results in Fig S2B. Single inhibitor treatment group decreased the NESTIN^+^SOX1^+^ cell numbers, respectively. Inhibitors combination group exhibited a more powerful repressive effect than single inhibitor group (Fig. S2B).

In a summary, E2 could increase positive cells number effectively, and two inhibitors combination could decrease the expression of ectoderm markers and IGF‐1 significantly than using them singly.

### E2 exposure promoted neural precursor cells differentiation through cross‐talk between IGF‐1 and ERβ

**Figure 4:**
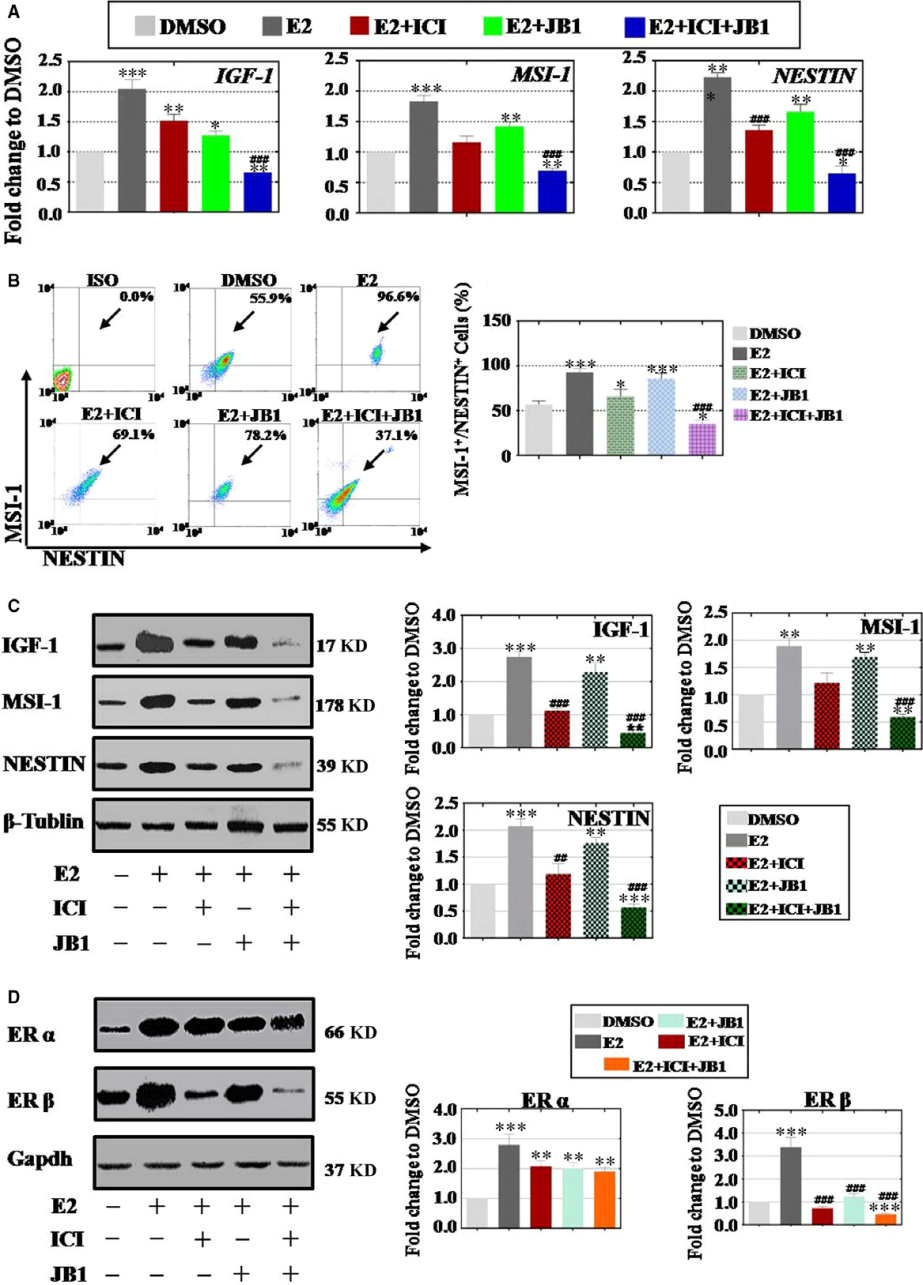
E2 induces neural precursor cells (NPCs) differentiation through insulin‐like growth factors (IGF)‐1 and oestrogen receptor (ER)β. (**A**) E2 treatment increased the expression of *IGF‐1* and NPC markers (*NESTIN* and *MSI‐1*), ICI or JB1 added reduced *IGF‐1* and NPC markers expression partly, inhibitors combination was repressed *IGF‐1* and NPC markers notably. (**B**) NESTIN and MSI‐1‐positive NPCs decreased upon E2 treatment, and ICI or JB1 separately supplied inhibited the cell population not wholly, antagonists combination (ICI and JB1) significantly reduced NESTIN and MSI‐1‐positive NPCs population. (**C**) Western blot results were as similar as qPCR and FACS that E2 advanced IGF‐1 and NPC markers expression, and single inhibitor just curbed their expression in some way, ICI plus JB1 significantly suppressed the protein level of IGF‐1 and NPC markers. (**D**) E2 induced the expression of ERβ higher than ERα, one or two inhibitors could repress the expression of ERβ more obviously than ERα, inhibitors combination prohibited the expression of ERβ more dominant than ERα, inhibitors combination group decreased the expression of ERβ more powerful than single inhibitor group at NPCs differentiation stage. Experiments were carried out at differentiation day 14, *n* = 3; Error bars indicate SD; *P < 0.05; **P < 0.01; ***P < 0.001 (compared with the DMSO group). #*P* < 0.05; ##*P* < 0.01; ###*P* < 0.001 (compared with the E2 group).

To detect the effect of E2 on the differentiation of hESCs into NPCs, as shown in Figure 1B, the culture system for directly induced neuron differentiation from hESCs was established. Here, groups of 0.1% DMSO, E2 (0.1 μM), E2 (0.1 μM) plus ICI (2 μM), E2 (0.1 μM) plus JB1 (1 μM) and E2 (0.1 μM) plus ICI (2 μM) with JB1 (1 μM) were employed. We handled qPCR, FACS and Western blot methods to test the level of mRNA and protein that involve in IGF‐1 and NPC markers (NESTIN and MSI‐1). As shown in Figure 4A, qPCR assay results demonstrated that E2 treatment showed positive effects on NPCs differentiation, significantly increased the expression of *IGF‐1* (reaching 205%), *NESTIN* (reaching 223%) and *MSI‐1* (reaching 183%). FACS assay results demonstrated that E2 also improved the NESTIN^+^MSI‐1^+^ NPCs production (reaching 92%) to that of DMSO group (reaching 56%). However, when antagonist ICI or JB1 was added into E2 group, respectively, the expression of *IGF‐1*,* NESTIN* and *MSI‐1* was down‐regulated to 151%, 135% and 116% in ICI group, 127%, 166% and 142% in JB1 group, compared to that of DMSO group. When ICI and JB1 added into E2 group together, we found that mRNA level of these genes was significantly repressed to *IGF‐1* (66%), *NESTIN* (65%) and *MSI‐1*(69%) in Figure 4A. Our further FACS assay implicated that the number of NPCs (NESTIN^+^MSI‐1^+^ cells) in E2 group was also increased at differentiation day 14, reached to 92% to that of DMSO group. Likewise, ICI or JB1 supplied singly could partially inhibit the positive effect of E2, and percentage of NESTIN^+^MSI‐1^+^ cells was 65% or 75%, respectively (Fig. 4B). However, when we added inhibitors combination (ICI plus JB1) into E2 group, the number of NESTIN^+^MSI‐1^+^ cells was decreased to 35% significantly (Fig. 4B). Protein level assay also showed the similar results, and E2 could improve the expression of IGF‐1, NESTIN and MSI‐1, respectively. Antagonist ICI or JB1 was supplied into E2 group individually that just inhibited the protein expression slightly to 192% (IGF‐1), 118% (NESTIN) and 122% (MSI‐1) in ICI group, and 152% (IGF‐1), 176% (NESTIN) and 169% (MSI‐1) in JB1 group to that of DMSO group. Nevertheless, E2 with antagonist mixture (ICI plus JB1) group manifested that the protein expression was significantly reduced to 45% (IGF‐1), 56% (NESTIN) and 59% (MSI‐1) (Fig. 4C). In E2 treatment group, the expression of ERα was elevated to 279%. Concurrently, the protein level of ERα was reduced slightly in response to 208% in ICI group, 201% in JB1 group and 190% in inhibitors combination group compared to that of DMSO group (Fig. 4D). In E2 treatment group, the expression of ERβ was elevated to 339%. Differently, the protein of ERβ was significantly decreased in response to 72% in ICI group, 124% in JB1 group and 46% in inhibitors combination group compared to that of DMSO group (Fig. 4D). In IGF‐1 treatment group, qPCR and FACS assay also demonstrated the results that are similar to E2 group. IGF‐1 improved gene expression and increased NESTIN^+^MSI‐1^+^ NPCs production. Single antagonist ICI or JB1 inhibited the gene expression and reduced the number of NESTIN^+^MSI‐1^+^ NPCs moderately. Inhibitors combination (ICI plus JB1) could totally reduce the gene expression level and NESTIN^+^MSI‐1^+^ NPCs production (Fig. S3A,B). Moreover, after IGF‐1 addition, the expression level of ERα was hard to change in ICI, JB1 or inhibitors combination group (Fig. S3C). Furthermore, the protein level of ERβ was significantly decreased in response to 136% in ICI group, 95% in JB1 group and 41% in inhibitors combination group compared to that of DMSO group (Fig. S3C). To avoid off‐target effects from inhibitor (ICI or JB1), the experiments of gene silencing with siRNA were executed in this paper. Cell samples were collected at day 14 to test by Western blot. First, protein level results showed that ERβ siRNA and IGF‐1 siRNA treatment significantly decreased the protein expression of IGF‐1 (45% and 29%) in Fig. S4Aa and ERβ (41% and 56%) in Figure S4Ab, respectively, that compared to control group. In E2 treatment group, the protein expression of IGF‐1 or ERβ was elevated to 280% or 318%, respectively (Fig. S4Ba,Bb). And then, E2 was added into IGF‐1 siRNA and ERβ siRNA groups, and the effect from E2 was significantly inhibited to 121% (IGF‐1) in ERβ siRNA group and 209% (IGF‐1) in IGF‐1 siRNA group compared to that of E2 group in Fig. S4Ba. The protein expression of ERβ was significantly decreased in response to 67% in ERβ siRNA group, and 135% in IGF‐1 siRNA group compared to that of E2 group in Fig. S4Bb at differentiation day 14.

In a word, E2 treatment promoted NPCs differentiation and antagonists combination (ICI plus JB1) inhibited this kind of effect availably.

### E2 exposure promoted DA neuron differentiation and function through cross‐talk between IGF‐1 and ERβ

**Figure 5:**
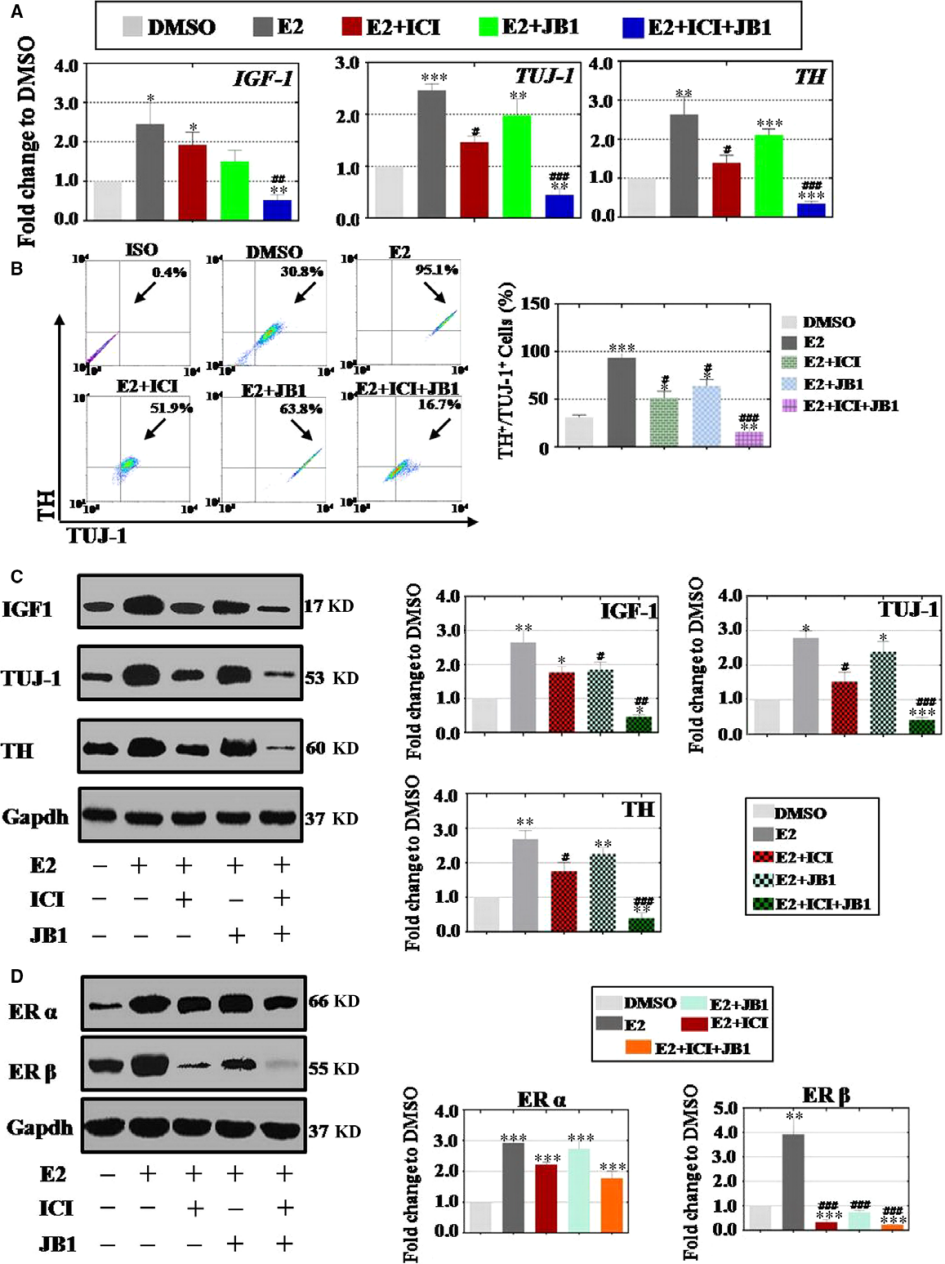
E2 induces dopamine (DA) neurons differentiation through insulin‐like growth factors (IGF)‐1 and oestrogen receptor (ER)β. (**A**) E2 treatment increased the expression of *IGF‐1* and DA neuron markers of *TH* and *TUJ‐1*, single inhibitor supplied decreased *IGF‐1*,* TH* and *TUJ‐1* expression partly, inhibitors combination repressed decreased *IGF‐1*,* TH* and *TUJ‐1* expression greatly. (**B**) FACS assay indicated tyrosine hydroxylase (TH)‐ and TUJ‐1‐positive DA neurons increased upon E2 treatment, one inhibitor supplied partially decreased the number of TH‐ and TUJ‐1‐positive DA neurons and inhibitors combination supplied completely inhibited the cell population. (**C**) Western blot assay revealed that the protein level of IGF‐1 and DA neuron markers (TH and TUJ‐1) was promoted after E2 treatment, single antagonist only repressed IGF‐1 and DA neuron markers in part and inhibitors combination supplied could completely inhibit the expression of above three proteins. (**D**) E2 activated the expression of ERβ higher than ERα at DA neurons differentiation stage, one or two inhibitors curbed the expression of ERβ stronger than ERα and inhibitors combination group decreased the expression of ERβ more powerful than single inhibitor group. Experiments were carried out at differentiation day 30, *n* = 3; Error bars indicate SD; *P < 0.05; **P < 0.01; ***P < 0.001 (compared with the DMSO group). ^#^ *P* < 0.05; ^##^ *P* < 0.01; ^###^ *P* < 0.001 (compared with the E2 group).

According to our previous reported protocol 14, DA neurons were differentiated from hESCs at day 30. Here, we explored whether E2 treatment affected the differentiation and function of DA neurons (Fig. 1C). At differentiation day 30, qPCR results showed that E2 increased expression of DA neuron markers (*TH* 263% and *TUJ‐1* 246%) and *IGF‐1* (245%). When ICI or JB1 was administrated into E2 group separately, mRNA level of these three genes was repressed incompletely, to *TH* (144% or 161%), *TUJ‐1* (156% or 178%) and *IGF‐1* (193% or 151%). However, E2 with inhibitors combination group caused a sharp decline in the expression of *TH* (35%), *TUJ‐1* (45%) and *IGF‐1* (53%) (Fig. 5A). Similarly, FACS analysis demonstrated that E2 could greatly elevate the number of TH^+^TUJ‐1^+^ cells (93%) at differentiation day 30 to that of DMSO group (31%), and then ICI or JB1 was separately supplied into E2 group that just inhibited this effect a little [TH^+^TUJ‐1^+^ cells (51% or 64%)]. Eventually, inhibitors combination group decreased the number of TH^+^TUJ‐1^+^ cells reaching 15% compared to that of DMSO group (Fig. 5B). Consistently, Western blot results showed that E2 exposure significantly increased the protein expression of TH (268%), TUJ‐1 (279%) and IGF‐1 (265%). Predictably, single ICI or JB1 was added into E2 group, and the expression of above genes just be inhibited partly. However, inhibitors combination repressed the three genes expression that were significantly dropped to 39% (TH), 42% (TUJ‐1) and 46% (IGF‐1) (Fig. 5C and Fig. S1). Oestrogen receptors expression were tested in our experiment. The assay data showed that E2 improved the expression of ERα (312%) and ERβ (392%) at differentiation day 30. Single ICI or JB1 treatment group curbed above effects slightly for ERα. Differently, protein level of ERβ was significantly repressed to 43% and 75% in single ICI or JB1 additional group. ICI plus JB1 combination group significantly decreased the expression of ERβ (23%), but not ERα (147%) (Fig. 5D). Parallel experiments in IGF‐1 treatment group were performed. At differentiation day 30, qPCR and FACS experiments also showed the similar results just like E2 group. First, E2 promoted the gene expression (*TH*,* TUJ‐1* and *IGF‐1*) and increased the number of TH^+^TUJ‐1^+^ cells (Fig. S3D,E). And then, when ICI or JB1 was administrated into IGF‐1 group separately, we found the gene expression and the number of positive TH^+^TUJ‐1^+^ cells were repressed incompletely (Fig. S3D,E). However, inhibitors combination (ICI and JB1) suppressed the gene expression and decreased the TH^+^TUJ‐1^+^ cells production completely (Fig. S3D,E). In our protein level assay, IGF‐1 could elevate ERα and ERβ expression. Nonetheless, single inhibitor (ICI or JB1) repressed ERβ expression partly, not ERα. Meanwhile, inhibitors combination decreased ERβ expression totally, but still not ERα (Fig. S3F).

**Figure 6:**
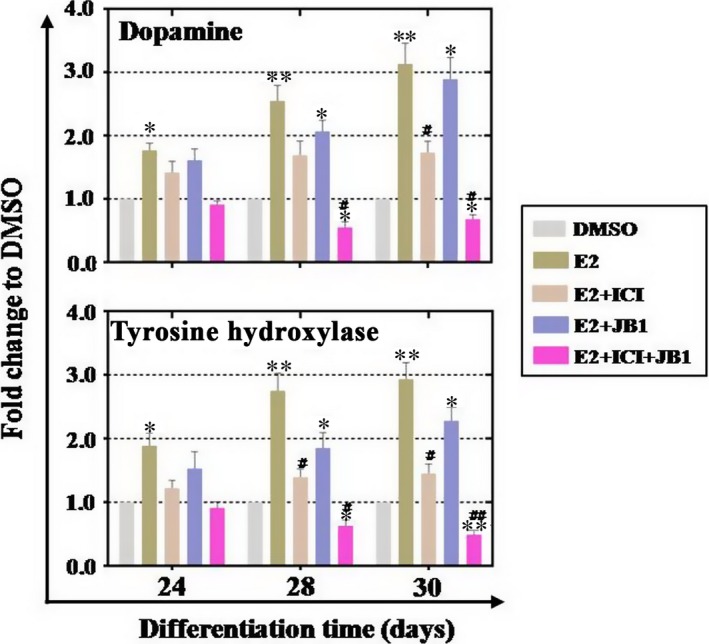
E2 promoted the secretion of dopamine neurotransmitter and tyrosine hydroxylase from human embryonic stem cells (hESCs)‐derived dopamine (DA) neurons. ELISA analysis indicated that hESCs‐derived DA neurons secreted dopamine and tyrosine hydroxylase. E2 administration gradually increased dopamine and tyrosine hydroxylase production (days 24, 28 and 30), single inhibitor just decreased the production of above two neurotransmitters partially, and inhibitors combination could completely reduce the creation of these two neurotransmitters. Experiments were carried out at differentiation day 30, *n* = 3; Error bars indicate SD; *P < 0.05; **P < 0.01; ***P < 0.001 (compared with the DMSO group). ^#^ *P* < 0.05; ^##^ *P* < 0.01; ^###^ *P* < 0.001 (compared with the E2 group).

DA neurons with function could secrete the neurotransmitter of TH and dopamine. We collected supernate to test which from differentiated DA neurons secrete TH and dopamine at days 24, 28, 30. Our ELISA assay results showed that E2 treatment significantly increased the production of dopamine (189%, 275% and 293%, according to days 24, 28 and 30) and TH (177%, 255% and 313%, according to days 24, 28 and 30) (Fig. 6). When ICI or JB1 was added separately, slightly reduced secretion of dopamine (reaching 228% and 145% left at day 30) and TH (reaching 289% and 173% left respectively at day 30) was observed in treatment group compared to that of E2 group (Fig. 6). When ICI and JB1 combination was added into E2 group, we found that the secretion of two neurotransmitters was significantly decreased, reaching to 91%, 63% and 49% left in dopamine test group (according to days 24, 28 and 30) and 121%, 145% and 143% left in TH test group (according to days 24, 28 and 30) (Fig. 6).

Summarily, E2 treatment promoted NPC differentiation and function, meanwhile, antagonists combination (ICI plus JB1) inhibited this kind of effect strikingly.

## Discussion

The results of the present study demonstrate that E2 plays a pivotal role in stimulating the DNA synthesis of mouse ES cells 22. Our findings also strongly suggest that E2 induces stimulation of hESCs proliferation. In the previous study, the serum concentration of E2 is 0.15 μM in the third trimester of pregnancy 8. Many studies have already elucidated that E2 with different concentrations enhance survival and differentiation in a multitude kinds of cell, such as neural cells and human bone marrow mesenchymal cells 23, 24. The present effects of E2 measured at 0.1 and 1 μM concentrations represent physiological actions; however, E2 with high concentration (10 μM) suppresses hESCs proliferation and induces apoptosis. Our results hint that distinct concentrations of E2 in pregnant women will disturb development of embryo. Thus, we choose appropriate concentration of E2 (0.1 μM) to operate the under experiment in hESCs.

Pioneering work showed that nucleic acid and protein level of ERα and ERβ was present in undifferentiated hESCs and hEBs 21. However, little is known about the involvement of ERs in the process of hESCs differentiation into DA neurons. According to our foregoing reported protocol 14, we define this protocol to three stages to investigate, EBs, NPCs and DA neurons stages (Fig. 1). In our study, we first proved that E2 can up‐regulate ectoderm markers and IGF‐1 in hEBs stage (Fig. 2). It may be attributed to the fact that ectoderm‐derived neuron system is the earliest system developed during human embryogenesis, in which human immune system has not been well established thus developing organ is easily affected by external factors. Considering that attenuated DA neurons may cause Parkinson's disease character, and it is reasonable that E2 level in pregnant woman may influence the development of embryo 25. The peak expression of local IGF‐1 is often spatially correlated with the active proliferation, development and growth of neural cells. In human, IGF‐1 as a kind of additive to increase the survival of mammalian embryos by increasing the proportion of preimplantation embryos that eventually become blastocysts 26. This effect would be disturbed in response to various factors such as heat shock, oxidative stress, tumour necrosis factor α and toxicity. Protective actions of IGF‐1 appear to be developmentally regulated.

Our previous study indicated that E2 could up‐regulate the expression of IGF‐1 during hESCs differentiation period 14. However, poorly understand that whether E2 can regulate hESCs differentiation into DA neurons and how to E2 work during this period. Previous research showed that oestrogen affects the proliferation and differentiation of neural stem cells (NSCs) via the ERs, probably in conjunction with other factors governing the development of NSCs 27. Results of the present study indicate that E2 promotes hESCs differentiation in NPCs and DA neurons stage in mRNA and protein level. Concurrently, IGF‐1 inhibitor (JB1) can partially reduce the expression of IGF‐1 and markers from these three stages (Figs 3, 4, 5). Subsequently, we further found that ER inhibitor (ICI) can also repress the E2 effect from these three stages incompletely (Figs 3, 4, 5). Therefore, we confirmed that the effect of E2 improves hESCs differentiation into DA neurons via regulating IGF‐1 and ER signal pathway.

Previous research indicated that E2 enhances neuronal differentiation of mouse ESC 28. Meanwhile, several lines of evidence support the cross‐talk between IGF‐1 and ER at different levels, such as in cardiovascular system 29, hepatic system 30 and central nervous system 31. Even so, it is still little known about that whether this interaction is present in process of hESCs differentiation. To address this issue further, inhibitors combination (ICI plus JB1) is used to clarify this question. During whole differentiation period, our mRNA and protein results revealed that the inhibitors combination exhibits stronger reverse effect than using them alone (Figs 3, 4, 5). Functional study also showed the similar results that inhibitors combination reduced the production of DA neurotransmitter greatly (Fig. 6). These current findings are consistent with results of the previous study that interactions of oestrogen and IGF‐1 have focused directly on neurons 32. Off‐target effects of inhibitors existed in experiments or clinical therapy now and then 33. To confirm whether inhibitor (ICI or JB1) treatment results were caused by off‐target effects, RNAi assay was performed at NPCs stage. The protein assay results revealed that IGF‐1 siRNA and ERβ siRNA transfection inhibited E2‐positive effects partly at NPCs stage (Fig. S4B). These results are consistent with previous protein assay results that used inhibitors (ICI or JB1) to prohibit this effect (Fig. 4C and 4B).

ERs are thought to be located in or near the plasma membrane, where they can access the mechanisms of signal generation. Document revealed that two types of ERs, ERα and ERβ, share the common feature of the nuclear receptor structure but are encoded by different genes located on different chromosomes, and in addition, they exhibit different brain distribution profiles 34. Thereby which receptor (ERα and ERβ) can interact with IGF‐1 is a key problem that needs to be clarified. At NPCs and DA neurons stage, we observed that inhibitor (ICI) significantly abolished the expression of ERβ in protein level, but not ERα (Figs 4D and 5D). These findings are similar to those from studies on ERβ mRNA expression, which were more than 10‐fold higher than ERα in hNPCs 27. Meanwhile, our findings are also supported by a recent study indicating that hESCs and hEBs express fivefold higher ERβ than ERα 35. Indeed, many works indicated that ERβ as a predominant receptor to regulate the development of various neuronal cells, such as serotonergic neurons 36 and oligodendrocytes 37. Earlier research also revealed that ERβ may combine with other genes to coordinate the development of NSCs 38. In the present study, we have found that inhibitors combination has ability to repress the protein level of ERβ stronger than one inhibitor in NPCs and DA neurons (Figs 4D and 5D). That means the cross‐talk between IGF‐1 and ERβ plays a vital role in hESCs differentiation into DA neurons. These findings are consistent with those previously reported and also support the idea that the ER and IGF‐1R signalling pathways are important for the development of NPCs 10, 32.

**Figure 7:**
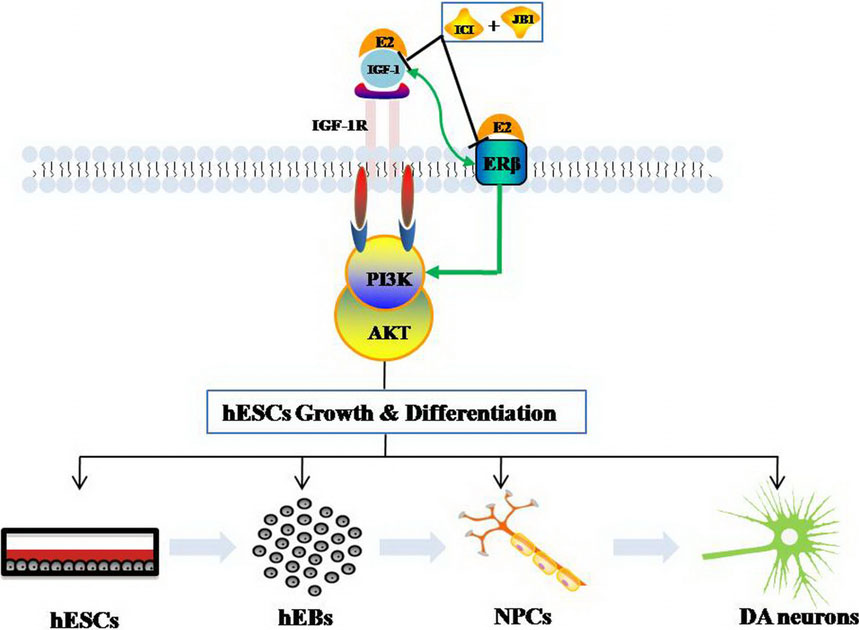
Proposed model for the interaction of insulin‐like growth factors (IGF)‐1 and oestrogen receptor (ER)β in human embryonic stem cells (hESCs) differentiation into dopamine (DA) neurons. During the process of hESCs differentiation into DA neurons, E2 could up‐regulate the expression of IGF‐1 and ERβ, ICI or JB1 had ability to repress IGF‐1 and ERβ, respectively. Thus, E2 enhanced hESCs differentiation into DA neurons through the cross‐talk between IGF‐1 and ERβ.

In conclusion, it is well known that E2 as a sex hormone and its effects play a key role in foetal development and growth 8. The results of the present study provide insight into the mechanism of E2 which promotes the hESCs differentiation into DA neurons by cross‐talk between IGF‐1 and ER. Furthermore, in oestrogen receptors, our present study revealed that ERβ is a crucial receptor subtype in this interaction signal pathway. Therefore, we suggested that E2 promotes neural cell differentiation mainly through IGF‐1/ERβ‐PI3K‐AKT signal pathway (Fig. 7). This discovery has important implication for understanding the molecular mechanisms by which factors promote neurogenesis in human period. Moreover, this discovery suggests that IGF‐1 may serve as a novel, safer, and efficacious therapeutic target to improve neurogenesis *in vivo* to sustain neurological function and renewal in foetus development.

## Conflict of interest

The authors declare no conflicts of interest.

## Supporting information


**Fig. S1** At day 30, E2 treatment promoted DA neurons differentiation from hESCs. Immunostaining experiments indicated DA neuron markers of TUJ‐1 and TH expressed.

**Fig. S2.** IGF‐1 exposure up‐regulated IGF‐1 and marker genes’ expression of ectoderm layers during hESCs differentiation period.

**Fig. S3.** IGF‐1 induces NPCs and DA neurons differentiation through IGF‐1 and ERβ.

**Fig. S4.** IGF‐1 siRNA or ERβ siRNA transfection down‐regulated IGF‐1 and ERβ expression at NPCs stage.
