# Supplementary figures and images for: 17β‐Oestradiol promotes differentiation of human embryonic stem cells into dopamine neurons via cross‐talk between insulin‐like growth factors‐1 and oestrogen receptor β
Source: J Cell Mol Med. 2017 Feb 28;21(8):1605–18. doi: 10.1111/jcmm.13090 (PMC5542902; doi:10.1111/jcmm.13090)

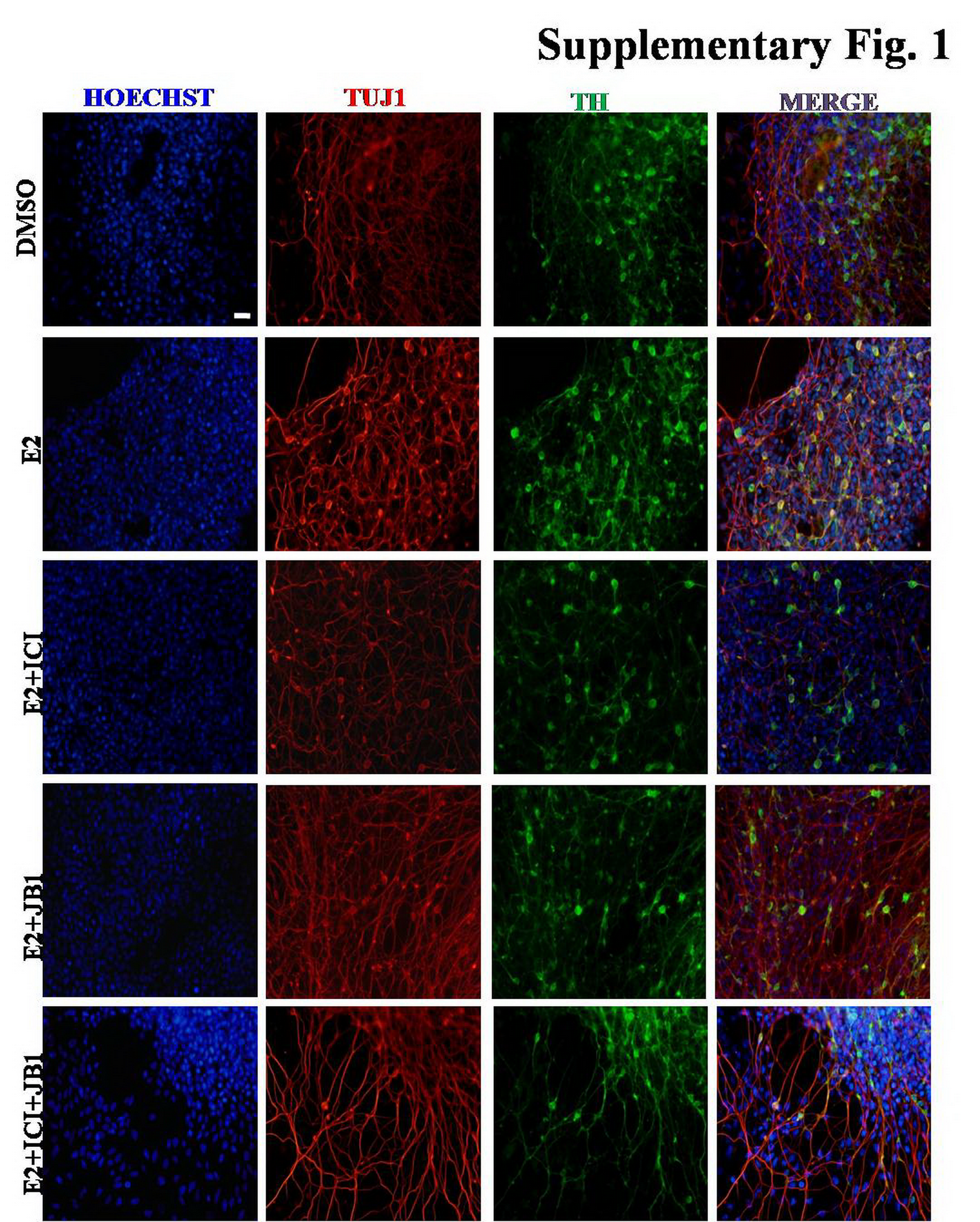

Supplement: Supplementary file 1 — Fig. S1 At day 30, E2 treatment promoted DA neurons differentiation from hESCs. Immunostaining experiments indicated DA neuron markers of TUJ‐1 and TH expressed. [file JCMM-21-1605-s001.tif]

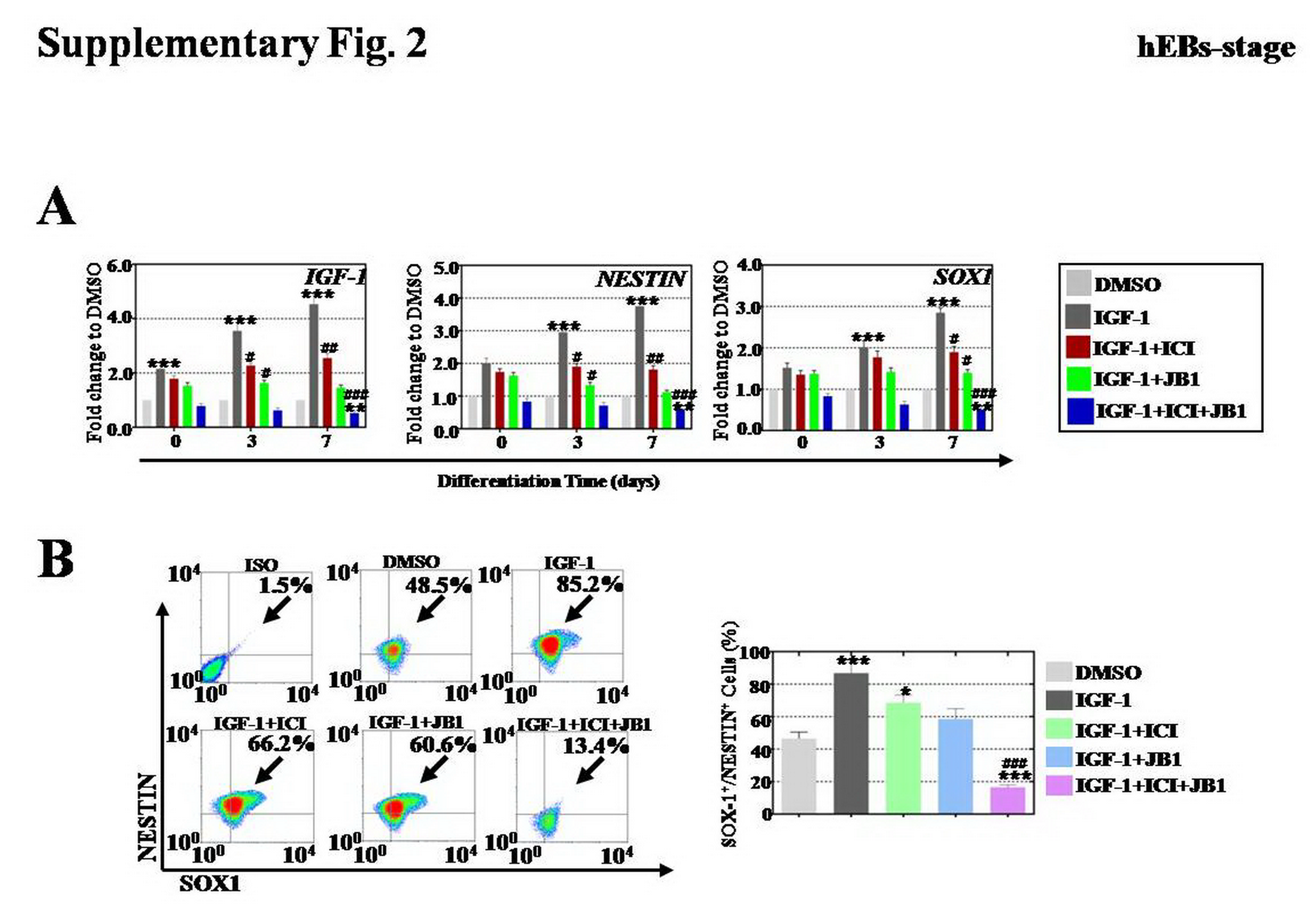

Supplement: Supplementary file 2 — Fig. S2. IGF‐1 exposure up‐regulated IGF‐1 and marker genes’ expression of ectoderm layers during hESCs differentiation period. [file JCMM-21-1605-s002.tif]

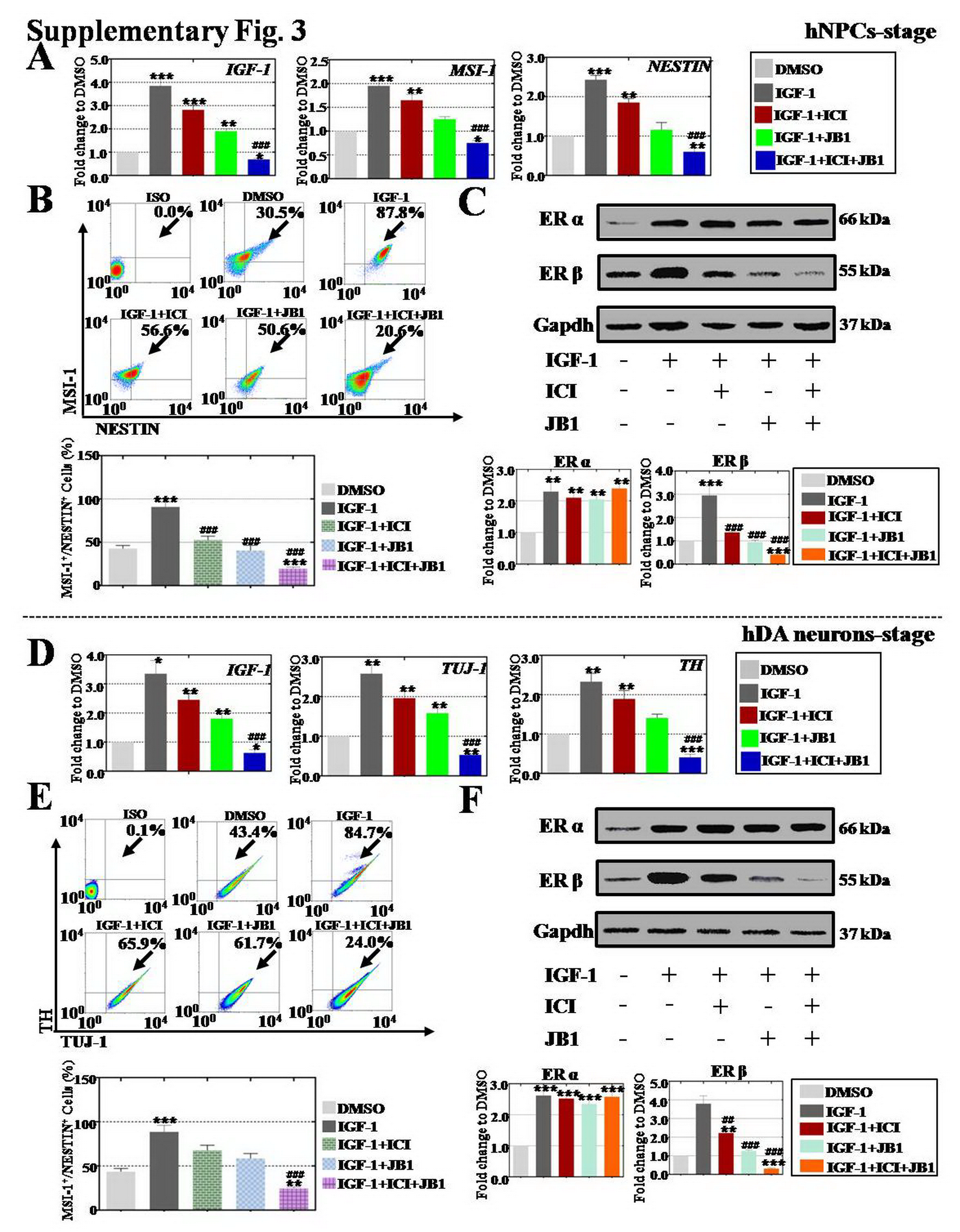

Supplement: Supplementary file 3 — Fig. S3. IGF‐1 induces NPCs and DA neurons differentiation through IGF‐1 and ERβ. [file JCMM-21-1605-s003.tif]

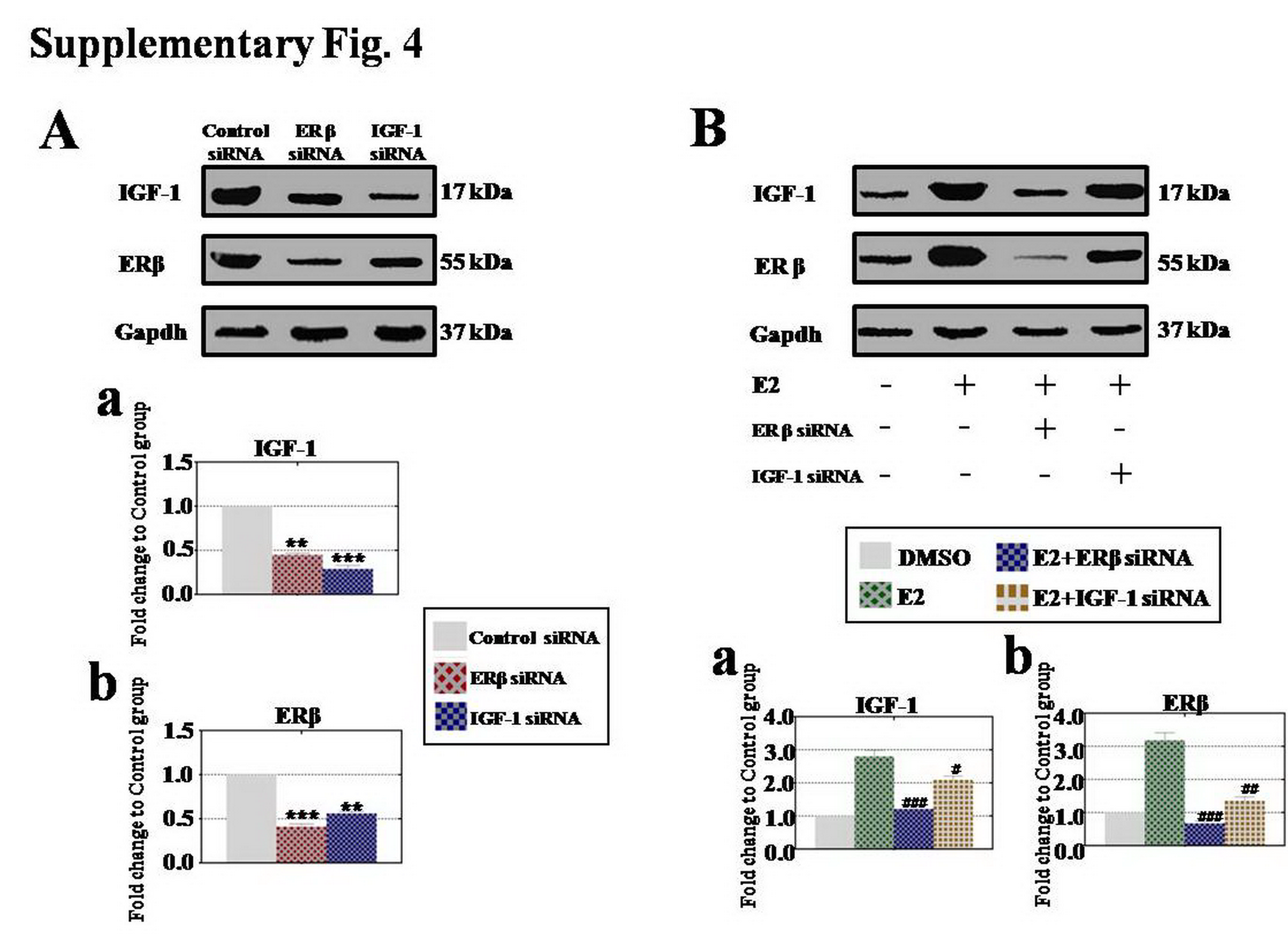

Supplement: Supplementary file 4 — Fig. S4. IGF‐1 siRNA or ERβ siRNA transfection down‐regulated IGF‐1 and ERβ expression at NPCs stage. [file JCMM-21-1605-s004.tif]
