# Supplementary material for: 17β‐Oestradiol promotes differentiation of human embryonic stem cells into dopamine neurons via cross‐talk between insulin‐like growth factors‐1 and oestrogen receptor β
Source: J Cell Mol Med. 2017 Feb 28;21(8):1605–18. doi: 10.1111/jcmm.13090 (PMC5542902; doi:10.1111/jcmm.13090)
Supplement: Supplementary file 5 [file JCMM-21-1605-s005.docx]

**Fig. S1** At day 30, E2 treatment promoted DA neurons differentiation from hESCs. Immunostaining experiments indicated DA neuron markers of TUJ-1 and TH expressed. Single inhibitor (JB1 or ICI) decreased the cells expressing TH and TUJ-1 lightly, and inhibitors combination application repressed the positive population strongly. Scar bar, 50 μm.

**Fig. S2.** IGF-1 exposure up-regulated IGF-1 and marker genes’ expression of ectoderm layers during hESCs differentiation period. **A.** *IGF-1*, *NESTIN* and *SOX-1* was measured by qPCR assay at three time-points. IGF-1 increased the expression of ectoderm markers of *NESTIN* (days 0, 3 and 7), *SOX1* (days 0, 3 and 7) and *IGF-1* (days 0, 3 and 7), while ICI or JB1 singly applied repressed *NESTIN* (days 3 and 7), *SOX1* (day 7) and *IGF-1* (days 3 and 7) expression partially. Inhibitors combination (ICI plus JB1) significantly reduced *NESTIN* (day 7), *SOX1* (day 7) and *IGF-1* (day 7) expression. **B.** At day 7, IGF-1, NESTIN and SOX-1 was measured by FACS assay. Results indicated that one inhibitor just curbed E2 effect slightly, inhibitors combination strongly decreased the population of NESTIN^+^SOX-1^+^ cells significantly, n=3; Error bars indicate SD. *, *p*<0.05**; *p*<0.01; ***, *p*<0.001 (compared with the DMSO group). #, *p*<0.05; ##, *p*<0.01; ###, *p*<0.001 (compared with the IGF-1 group)

**Supplementary Fig. S3. IGF-1 induces NPCs and DA neurons differentiation through IGF-1 and ERβ. A.** IGF-1 treatment increased the expression of *IGF-1* and NPC markers (*NESTIN* and *MSI-1*), ICI or JB-1 added reduced *IGF-1* and NPC markers expression partly, inhibitors combination were repressed *IGF-1* and NPC markers notably. **B.** NESTIN- and MSI-1-positive NPCs decreased upon IGF-1 treatment, and ICI or JB1 separately supplied inhibited the cell population not wholly, antagonists combination (ICI and JB1) significantly reduced NESTIN- and MSI-1-positive NPCs population. **C.** IGF-1 induced the expression of ERβ higher than ERα, one or two inhibitors could repress the expression of ERβ more obviously than ERα, inhibitors combination prohibited the expression of ERβ more dominant than ERα, inhibitors combination group decreased the expression of ERβ more powerful than single inhibitor group at NPCs differentiation stage. Experiments were carried out at differentiation day 14. **D.** IGF-1 treatment increased the expression of *IGF-1* and DA neuron markers of *TH* and *TUJ-1*, single inhibitor supplied decreased *IGF-1*, *TH* and *TUJ-1* expression partly, inhibitors combination repressed decreased *IGF-1*, *TH* and *TUJ-1* expression greatly. **E.** FACS assay indicated TH- and TUJ-1-positive DA neurons increased upon E2 treatment, one inhibitor supplied partially decreased the number of TH- and TUJ-1-positive DA neurons, and inhibitors combination supplied completely inhibited the cell population. **F.** IGF-1 activated the expression of ERβ higher than ERα at DA neuron differentiation stage, one or two inhibitors curbed the expression of ERβ stronger than ERα, inhibitors combination group decreased the expression of ERβ more powerful than single inhibitor group. Experiments were carried out at differentiation day 30, n=3; Error bars indicate SD; *, *p* < 0.05; **, *p* < 0.01; *** *p* < 0.001 (compared with the DMSO group). #, *p* < 0.05; ##, *p*<0.01; ###, *p*<0.001 (compared with the IGF-1 group). I= ICI; J=JB1.

**Supplementary Fig. S4. IGF-1 siRNA or ERβ siRNA transfection down-regulated IGF-1 and ERβ expression at NPCs stage.** IGF-1 and ERβ were measured by Western blot assay at NPCs stage. **Aa**, IGF-1 siRNA or ERβ siRNA transfection significantly reduced the expression of IGF-1. **Ab**, the expression of ERβ was curbed after IGF-1 siRNA or ERβ siRNA transfection respectively. **Ba**, IGF-1 siRNA or ERβ siRNA transfection significantly decreased the expression of IGF-1 in E2 treatment group. **Bb**, In E2 treatment group, the expression of ERβ was inhibited after IGF-1 siRNA or ERβ siRNA transfection respectively. Error bars indicate SD. *, *p*<0.05**; *p*<0.01; ***, *p*<0.001 (compared with the control group). #, *p*<0.05; ##, *p*<0.01; ###, *p*<0.001 (compared with the E2 group)
